# Mechanism of Apoptosis Induction by Mycoplasmal Nuclease MGA_0676 in Chicken Embryo Fibroblasts

**DOI:** 10.3389/fcimb.2018.00105

**Published:** 2018-04-04

**Authors:** Peng Li, Jian Xu, Hong-mei Rao, Xia Li, Yun-ke Zhang, Fei Jiang, Wen-xue Wu

**Affiliations:** ^1^Key Laboratory of Animal Epidemiology and Zoonosis, College of Veterinary Medicine, China Agricultural University, Beijing, China; ^2^Institute of Animal Husbandry and Veterinary Medicine, Beijing Academy of Agricultural and Forestry Sciences, Beijing, China; ^3^Veterinary Diagnostic Laboratory, China Animal Disease Control Center, Beijing, China

**Keywords:** mycoplasmal nuclease MGA_0676, apoptosis, endocytosis, NEDD8-activating enzyme E1 regulatory subunit, activation of NF-κB

## Abstract

MGA_0676 has been characterized as a *Mycoplasma gallisepticum* nuclease that can induce apoptosis of chicken cells. However, the mechanism by which MGA_0676 induces apoptosis has remained unclear. In this study, we evaluated MGA_0676-induced apoptosis and internalization in immortalized chicken embryo fibroblasts (DF-1) and cancer cell lines. The internalization of MGA_0676 was proven through caveolin-mediated endocytosis by blocking the endocytosis with specific inhibitors or with siRNA. We identified the Thif domain of NEDD8-activating enzyme E1 regulatory subunit (NAE) in DF-1 as the target region interacting with the SNC domain of MGA_0676. The interaction between the Thif and SNC domains was observed co-located in the perinuclear and nuclear of DF-1. We found that the interaction between NAE and MGA_0676 increased the ability of apoptosis and accelerated the process of cullin neddylation in DF-1 cells, in turn activating NF-κB. This resulted in the observed aggregation of NF-κB in the nuclei of DF-1 cells. Moreover, the apoptosis induced by MGA_0676 decreased significantly when NF-κB was inhibited by siRNA or BAY 11-7082 or when NAE was silenced by siRNA. Overall, our results demonstrate that MGA_0676 is internalized through caveolin-mediated endocytosis, interacts with SNC-dependent Thif to accelerate the process of cullin neddylation and activates NF-κB in DF-1 cells, ultimately playing a key role in apoptosis in chicken cells. Our results indicate MGA_0676 constitutes a critical etiological virulence factor of the respiratory disease caused by *M. gallisepticum*. This study also opens a venue to investigate MGA_0676 as a potential candidate as pro-apoptotic drug in cancer studies.

## Introduction

Microbes have been reported to mediate pathogenicity in host cells by utilizing their virulence proteins (Fitzgerald et al., 2006; Humphrys et al., 2013; Krachler and Orth, 2013). Many of these virulence-associated proteins have also been found to play a significant role in cytotoxicity through their interaction with host cell proteins (Casadevall and Pirofski, 2001; Sourjik and Berg, 2004; Chen et al., 2011). Previous reports have shown that proteins of microbes with nuclease activity are cytotoxic to host cells, and are directly related to their pathogenicity (Pediaditakis et al., 2012; Milner et al., 2014; Sacco et al., 2014). Nucleases are expressed by many different pathogenic microbes and have been shown to play a role in creating favorable nutrient-rich environments (Cuatrecasas et al., 1967; Ma and Goodridge, 1992; Suh and Benedik, 1997). Recently, it has been demonstrated that many pathogenic microbes possess diverse nucleases with biological functions related to the metabolism of nitrogen, phosphate and carbohydrates, as well as their survival and virulence (Suciu and Inouye, 1996; Dominski, 2007; Lennon, 2007). Other studies have indicated that microbial nucleases degrade neutrophil extracellular traps (NETs) and evade the host cell innate immune responses (Papayannopoulos and Zychlinsky, 2009; Thammavongsa et al., 2013; Toid, 2014). Furthermore, some bacteria possess more than one kind of nucleases, which are vital to virulence and contribute to a wide variety of cellular biological processes, such as inducing host cell death, repairing DNA damage, and evading immune clearance from host tissues (Rottem, 2003; Amundsen et al., 2008; Delaney et al., 2012; Derré-Bobillot et al., 2013; Shields et al., 2013). Due to their extremely limited biosynthetic capabilities, *Mycoplasma* adopts a parasitic lifestyle in order to obtain their nutritional needs from host cells (Chung et al., 2010; Fan et al., 2010; Großhennig et al., 2013). Without the ability to synthesize *de novo* purine and pyrimidine bases, *Mycoplasma* has to salvage nucleotide bases to produce nucleotide precursors (Wanga et al., 2014). However, these salvage pathways result in a series of pathological cellular processes, such as inflammation and apoptosis (Razin, 1999; Nakhyung, 2009). Numerous intracellular, extracellular and, particularly, membrane-associated nucleases have been reported in different *Mycoplasma* species, many of which are implicated in host pathogenicity and cytotoxicity through the degradation of nucleotides and induction of apoptosis-like cell death (Pollack and Hoffmann, 1982; Minion et al., 1993; Paddenberg et al., 1998). Some membrane-associated nucleases have been shown to have a SNC region and able to translocate into cells, a process followed by cytotoxic effects and induction of apoptosis, such as MPN133 in *M. pneumoniae*, mhp379 in *M. hyopneumoniae*, and MG186 in *M. genitalium* (Schmidt et al., 2007; Li et al., 2010; Somarajan et al., 2010). Therefore, it is worthwhile to examine the biological properties and mechanisms of mycoplasmal membrane-associated nucleases.

Previously, we found that *M. gallisepticum* MGA_0676 was a Ca^2+^-dependent cytotoxic nuclease containing a SNC region similar to other mycoplasmal nucleases, which could translocate into chicken cells and induce apoptosis in a SNC-dependent manner (Xu et al., 2015). However, the mechanism by which MGA_0676 induced apoptosis remained unclear. Nuclear factor-kappa B (NF-κB) is a very important molecule associated with many signaling pathways, but few studies have been made to investigate the relationship between NF-κB and apoptosis. To evaluate these mechanisms, in the present study we show that MGA_0676 internalizes through caveolin-mediated endocytosis, interacts with Thif-dependent SNC, accelerating the process of cullin neddylation and activating NF-κB in DF-1 cells, ultimately inducing apoptosis. In addition, we also show that MGA_0676 may be an important etiological virulence factor of the respiratory disease caused by *M. gallisepticum*, and that it may be involved in the immunosuppression of the infected birds.

## Materials and methods

### Bacterial strains, plasmids, and DNA manipulations

*M. gallisepticum* from the BJ44T strain (CVCC350, preserved in China Veterinary Culture Collection Center, Beijing, China) were grown in PPLO medium (BD, Franklin Lakes, NJ, USA) as described previously (Xu et al., 2015). *Escherichia coli* (*E. coli*) T1 (Invitrogen, Carlsbad, CA, USA) and *E. coli* BL21(DE3) pLysS competent *E. coli* (TransGen Biotech, Beijing, China) were grown in Luria–Bertani (LB) broth and used to clone and express *M. gallisepticum* nuclease (MGA_0676, AE015450.2). Vectors pGEX-6p-1, pET28a, pEGF-N1, pCMV-HA-tag plamid, and pCMV-Myc-tag plamid (Novagen, Darmstadt, Germany) were used for DNA manipulations.

### Cell lines, proteins, antibodies, and reagents

Immortal chicken embryo fibroblasts (DF-1) and human embryonic kidney 293T cells (HEK293T) were obtained from ATCC (American Type Culture Collection, Manassas, VA, USA). All cells were cultured in Dulbecco's modified Eagle medium (DMEM, Invitrogen, Grand Island, NY, USA) supplemented with 10% fetal bovine serum (FBS) in a 5% CO_2_ incubator. All restriction enzymes were purchased from New England Biolabs (Ipswich, MA, USA). Annexin V/PI apoptosis assay kits were purchased from BD (Franklin Lakes, NJ, USA). Anti-GST polyclonal antibody, anti-GFP polyclonal antibodies were obtained from Santa Cruz Biotechnology (Dallas, TX, USA). Rabbit polyclonal anti-clathrin-1, anti-cholera antibodies anti-transferrin antibody, and anti-cleaved caspase 3 antibodies were obtained from Abcam (Cambridge MA, USA). Anti-HA monoclonal antibodies, anti-Myc antibodies, anti-Rela antibodies, anti-IκBα antibodies, anti p-IκBα antibodies, and β-actin antibodies were obtained from Abclonal Inc. (Cambridge MA, USA). Mouse anti-NAE polyclonal antibody was prepared with purified recombinant NAE protein according to a standard molecular biology technique (Xu et al., 2015). Mouse anti-MGA_0676 monoclonal antibody was prepared according to a previously reported standard protocol (Fu et al., 2014). Alexa Fluor 555-conjugated phalloidin (red) and Lipofectamine® LTX DNA transfection reagents were purchased from Invitrogen. Human holo-transferrin (Tf), cholera toxin-FITC, monodansylcadaverine (MDC), and filipin were purchased from Sigma-Aldrich (Louis, MO, USA). All other chemicals reagents used in the study were of analytical grade.

### Computer-assisted sequence analysis

The *M. gallisepticum* MGA_0676 sequence (MGA_0676) was downloaded from the UniProtKB database at http://www.uniprot.org/uniprot/C0SKM0. The NEDD8-activating enzyme E1 regulatory subunit (NAE, NP_001006129.1) of DF-1 cells was downloaded from the NCBI database (http://www.ncbi.nlm.nih.gov/protein/57524906). Domains of MGA_0676 and NAE were analyzed using the PROSITE database (http://www.expasy.ch/tools/scanprosite/). Prediction of the signal peptide cleavage sites in MGA_0676 was performed using SignalP Server (http://www.cbs.dtu.dk/services/SignalP/). Sequence alignment analysis and three-dimensional (3D) structure modeling were performed using ESPript 2.2 (http://espript.ibcp.fr/ESPript/cgi-bin/ESPript.cgi) and homology remodeling tools (http://swissmodel.expasy.org/workspace/index). Prediction of the interactions between proteins was done using the Hexserver tool (http://hexserver.loria.fr/). Software programs, such as DNAstar, DNAMAN, and Primer Premier 5, were also used in the analyses.

### Preparation of recombinant proteins

Recombinant His-tag MGA_0676 (rMGA_0676) and recombinant His-tag MGA_0676^Δ*SNC*^ (rMGA_0676^Δ*SNC*^) were prepared as described previously (Xu et al., 2015). Recombinant GST-tag MGA_0676 was prepared with vector pGEX-6p-1 and recombinant His-tag NAE was prepared with Vector pET28a. All proteins were purified using standard molecular biology techniques. Protein concentrations were determined by the Bradford protein assay using bovine serum albumin (BSA) as a standard. Nuclease activity of rMGA_0676 and rMGA_0676^Δ*SNC*^ was detected as described previously (Xu et al., 2015).

### Construction of plasmids

The MGA_0676 gene of *M. gallisepticum* was PCR amplified from the *M. gallisepticum* strain BJ44T chromosomal DNA. After changing the TGA encoded tryptophan codons using specific primers, the *M. gallisepticum* MGA_0676 gene was subcloned in the pEGF-N1 and pCMV-HA expression vectors. The plasmid was then introduced into *E. coli* T1 and extracted using a Qiagen plasmid kit. MGA_0676^Δ*SNC*^ (MGA_0676 without the SNC region) was amplified by OE-PCR using specific primers and construction of recombinant plasmid (pEGF-N1-MGA_0676^Δ*SNC*^ and pCMV-HA-MGA_0676^Δ*SNC*^) was performed as described previously (Xu et al., 2015).

The NEDD8-activating enzyme E1 regulatory subunit (NAE, 57524906) was cloned from the cDNA of DF-1 cells using specific primers (Table S1). The pCMV-Myc-NAE and pCMV-Myc-NAE^Δ*Thif*^ (NAE without Thif region) expression plasmids were constructed using standard molecular biology techniques. All the primers were synthesized by Sangon Biotech (Beijing, China) (Table S1).

### Detection of rMGA_0676 internalization in cells

For immunofluorescence antibody assays (IFA), HepsG2, A549, Hela, and 293A cells were treated with rMGA_0676 or rMGA_0676^Δ*SNC*^ (40 μg/ml) for 24 h at 37°C. DF-1 cells treated with recombinant proteins were used as positive controls. PBS-treated cells were used as negative controls. After incubation, cells were fixed with 4% paraformaldehyde, permeabilized with 0.25% TritonX-100 for 5–10 min, blocked with 3% bovine serum albumin, and incubated with mouse anti-His monoclonal antibody overnight at 4°C. Cells were then incubated with FITC-conjugated goat anti mouse IgG antibody (green) and cellular F-actin was stained with Alexa Fluor 555-conjugated phalloidin (red). Nuclei were counterstained with DAPI (blue). Cell samples were examined with a laser confocal scanning microscope (Leica, Saarbrücken, Germany).

DF-1 cells were treated with rMGA_0676 at concentrations of 20, 40, and 80 μg/ml for 24 h at 37°C to study the dose and time-dependence internalization of rMGA_0676. DF-1 cells were also treated with rMGA_0676 (40 μg/ml) at 37°C for 12, 24, and 36 h. All treated samples underwent IFA, as described above.

DF-1 cells were treated with filipin (0, 1.5, 3.75, 7.5 μM) and monodansylcadaverine (MDC) (0, 50, 100, or 200 μM) followed by rMGA_0676 for 24 h at 37°C to detect the inhibition of rMGA_0676 internalization. The samples underwent IFA and were examined with fluorescence microscopy. DF-1 cells were also pretreated with small interfering RNA to inhibit expression of caveolin or clathrin, and treated with rMGA_0676 for 24 h at 37°C, then analyzed by IFA, and observed using fluorescence microscopy.

### RNA interference (RNAi) knockdown of NAE, caveolin, clathrin, and NF-κB

SiRNAs, designed by Genepharma (Shanghai, China), were used to knock down NAE, caveolin, clathrin, and NF-κB in DF-1 cells. The siRNA sequences for targeting proteins in DF-1 cells were shown in Table S1. Cells were transfected with siRNA using RNAiMAX reagent, according to manufacturer's instructions (Invitrogen). Analysis was performed with western blots. Knockdown of NAE in DF-1 cells for evaluation of impact on apoptosis and the activation of NF-κB under different conditions. Knockdown of caveolin and clathrin in DF-1 cells for evaluation of internalization of rMGA_0676 under rMGA_0676 treated. Knockdown of NF-κB in DF-1 cells for evaluation of impact on activation of NF-κB under treated conditions.

### Apoptosis assays

Cells were cultured in six-well flat-bottomed plates and exposed to rMGA_0676 or rMGA_0676^Δ*SNC*^ (40 μg/ml) for 24 h to determine whether rMGA_0676 and/or rMGA_0676^Δ*SNC*^ induced apoptosis in cells. PI positive cells were then analyzed using the Annexin V-FITC/PI apoptosis detection kit, according to manufacturer's instructions.

Cells were cultured in six-well flat-bottomed plates and exposed to rMGA_0676 (40 μg/ml), MGA_0676^Δ92−221^ (40 μg/ml), or *M. gallisepticum* (MOI = 10) for 24 h to determine whether rMGA_0676 or *M. gallisepticum* induced apoptosis after NAE knockdown or treatment with NF-κB siRNA. Apoptotic cells were analyzed using the Annexin V-FITC/PI apoptosis detection kit, according to manufacturer's instructions. Apoptotic DF-1 cells were analyzed by flow cytometry. The above experiments were carried out in triplicate and the data were analyzed using SPSS software.

### Pull down, co-immunoprecipitation, and western blot analysis

For the pull down, DF-1 cells were seeded on six-well plates, cultured for 24 h, and then treated for 24 h with rGST-MGA_0676 (40 μg/ml); rGST was used as a negative control. DF-1 cells were also transfected with pEGF-N1-MGA_0676 for 24 h; pEGF-N1 was used as a negative control. All cell lysate samples were prepared with a non-denaturing lysis buffer. The pull down technology was performed with a detection kit, according to manufacturer's instructions.

For co-immunoprecipitation, HEK293T cells or DF-1 cells were seeded on plates and cultured for 24 h before co-transfection with pCMV-HA-MGA_0676, pCMV-HA-MGA_0676^Δ*SNC*^, pCMV-Myc-NAE, and pCMV-Myc-NAE^Δ*Thif*^; empty vectors were used as controls. Twenty-four hours after transfection, cell lysates were prepared using a non-denaturing lysis buffer. Cell lysates were incubated with 5 μg of anti-HA antibody at 4°C for 2 h and then mixed with 25 μl of a 50% slurry of protein A/G plus agarose and incubated for another 2 h. Beads were washed three times with lysis buffer and boiled with 6×SDS loading buffer for 5 min. The samples were fractionated by electrophoresis on 12% SDS-polyacrylamide gels and resolved proteins were transferred onto polyvinylidene difluoride membranes. After blocking with 5% skim milk, the membranes were incubated with either anti-HA or anti-Myc antibodies, followed by an appropriate horseradish peroxidase-conjugated secondary antibody. For the endogenous NAE assay, DF-1 cells were transfected with pCMV-HA-MGA_0676 or with an empty vector. Twenty-four hours after transfection, cell lysates were subjected to immunoprecipitation with anti-HA antibody and immunoblotted with anti-NAE or anti-FLAG antibodies. For the *M. gallisepticum* MGA_0676 and endogenous NAE assays, DF-1 cells were infected with *M. gallisepticum* (MOI = 10) for 36 h and the cell lysates were subjected to immunoprecipitation with anti-MGA_0676 antibody and immunoblotting with anti-NAE.

### Confocal laser scanning microscopy assays

HEK293T or DF-1 cells were seeded on cover slips in 24-well plates and cultured for 18–24 h, for overexpression of pCMV-HA-MGA_0676 and pCMV-Myc-NAE. The two plasmids were then co-transfected with LTX reagents. For overexpression of MGA_0676, DF-1 cells were treated only with rMGA_0676 (40 μg/ml) or transfected with pCMV-HA-MGA_0676. Twenty-four hours after transfection or treatment, cells were fixed with 1% paraformaldehyde, permeabilized with 0.1% TritonX-100 for 3–5 min and blocked with 3% bovine serum albumin. For co-localization of HA-MGA_0676 and Myc-NAE in DF-1 cells, the slides were incubated with mouse anti-HA antibody and rabbit anti-Myc antibody for 1.5 h at 37°C. For co-localization of HA-MGA_0676 and endogenous NAE in DF-1 cells, the slides were incubated with mouse anti-HA antibody and rabbit anti-NAE antibody for 1.5 h at 37°C. For co-localization of rMGA_0676 and endogenous NAE in DF-1 cells, the slides were incubated with mouse anti-His antibody and rabbit anti-NAE antibody for 1.5 h at 37°C. For co-localization of rMGA_0676 and caveolin, the sections were incubated with mouse anti-His antibody and rabbit anti-caveolin antibody for 1.5 h at 37°C. Sections were then incubated with FITC-conjugated goat anti-mouse IgG antibody (green) and rhodamine (tetramethyl rhodamine isocyanate [TRITC])-conjugated goat anti-rabbit antibody (red). Nuclei were counterstained with DAPI (blue).

### Detection of NF-κB activity

To detect NF-κB activity, DF-1 cells were seeded on six-well plates and cultured for 24 h, then treated with rMGA_0676 at concentrations of 0, 20, 40, or 80 μg/ml for 24 h. TNF-α treated cells were used as positive control. Cell lysates were prepared using a non-denaturing lysis buffer, and boiled with 6×SDS loading buffer for 10 min. The samples underwent immunoblotting (according to the manufacturer's instructions) for detection of Rela in the nucleus and IκBα and p-IκBα in the cytoplasm. PCNA was used as the nucleus internal reference and β-actin was used as the cytoplasm internal reference. Translocation of Rela was also detected in DF-1 cells. DF-1 cells were seeded on 24-well plates, cultured for 18–24 h and then treated with either rMGA_0676 (40 μg/ml) or *M. gallisepticum* (MOI = 10) for 24 h. Cells then underwent IFA, as previously described, after incubation with mouse anti-Rela antibody for 1.5 h at 37°C, followed by incubation with FITC-conjugated goat anti-mouse IgG antibody (green) and cellular F-actin staining with Alexa Fluor 555-conjugated phalloidin (red). Nuclei were counterstained with DAPI (blue). Cells were then observed with a laser confocal scanning microscope (Leica). DF-1 cells were treated with rMGA_0676 (40 μg/ml) or rMGA_0676^Δ*SNC*^ (40 μg/ml) for 24 h as described above to determine whether NF-κB is activated via interaction of MGA_0676 and NAE. DF-1 cells with silenced NAE were also treated with rMGA_0676 (40 μg/ml), and NC (unrelated RNA oligo) was used as negative control. The samples were analyzed as described above.

### Detection of activation of cullin neddylation in DF-1 cells

To detect activation of cullin neddylation, DF-1 cells were seeded on six-well plates and cultured for 24 h, then treated with rMGA_0676 at concentrations of 0, 20, 40, or 80 μg/ml for 24 h. Then cell lysates were prepared for western blot to detect the expression of cull-NEDD8 based on the anti-chicken cull antibody or anti-NEDD8 antibody.β-actin was used as internal reference. DF-1 cells were seeded on six-well plates and cultured for 24 h, then treated with MLN4924 (500 ng/l) for 12–18 h or MGA_0676 at concentrations of 40 μg/ml for 24 h. Then cell lysates were prepared for western blot to detect expression of cull-NEDD8 and NF-κB activity as described above. β-actin was used as internal reference.

### Statistical analysis

All the tests were triplicated, and the statistical differences between treated and control groups were determined and analyzed by analysis of variance (ANOVA) using SPSS software, version 18.0 (SPSS, Chicago, IL, USA), and the graphs were made using GraphPad Prism 5.0. “ns” represented no significant differences (*P* > 0.05). “^*^” represented statistically differences (*P* < 0.05), “^**^” represented statistically significant differences (*P* < 0.01), “^***^” represented statistically significant differences (*P* < 0.001).

## Results

### Caveolin-mediated endocytic mechanisms are implicated in internalization of rMGA_0676

Internalization of rMGA_0676 was determined by incubating chicken embryo fibroblasts (DF-1 cells) with different doses of recombinant nuclease at 37°C for 24 h. Initial determinations of the mechanism and internalization rates were shown in Figure S1. Internalization of rMGA_0676 by DF-1 cells increased with nuclease concentrations (Figure S1A). Internalization rates were also determined by incubating DF-1 cells with rMGA_0676 (40 μg/ml) at different times, and were found to increase with incubation times (Figure S1B).

**Figure 1:**
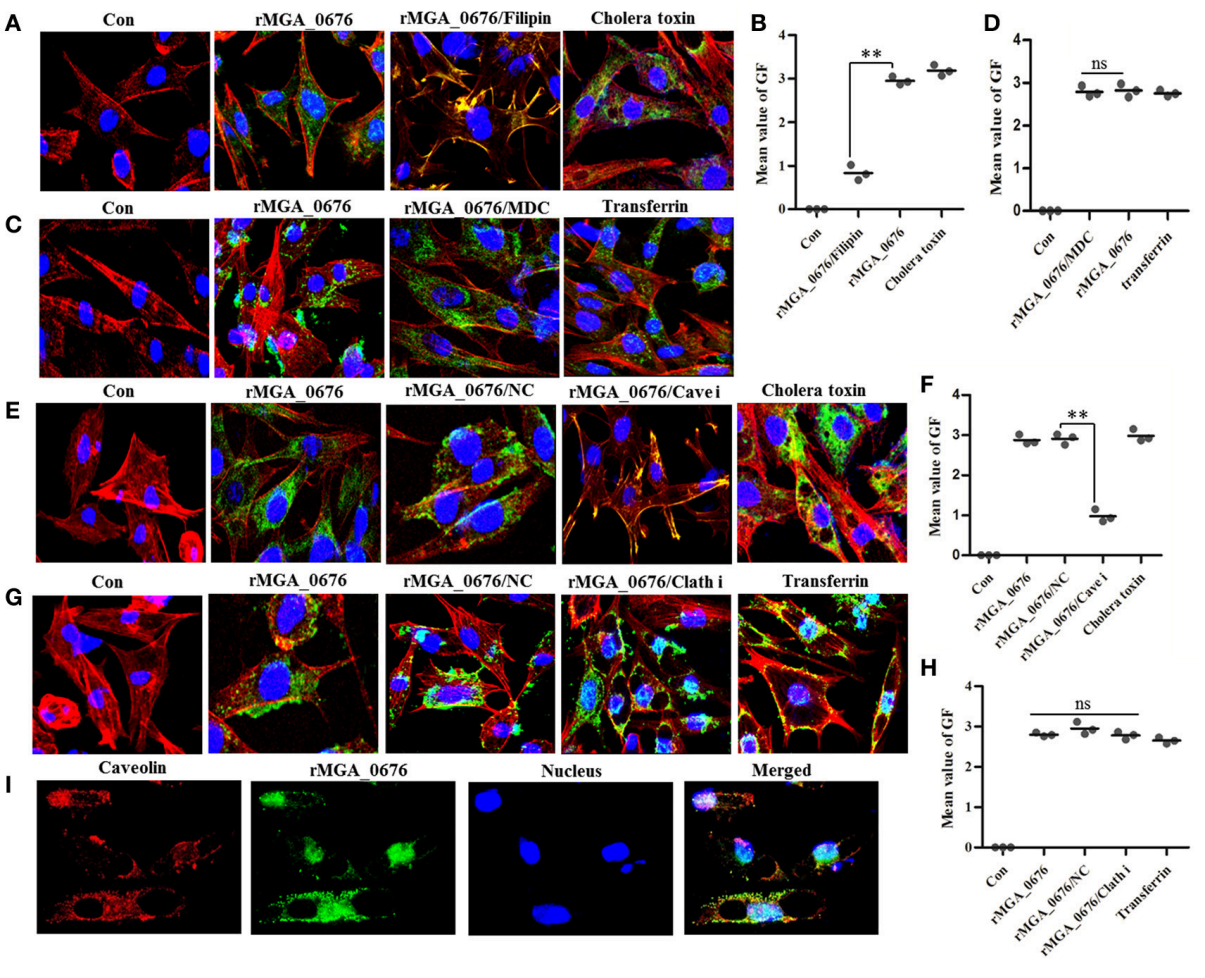
Caveolin-mediated endocytic mechanisms are implicated in internalization of rMGA_0676. **(A)** Internalization of rMGA_0676 was blocked by caveolin-mediated endocytic inhibitor filipin. DF-1 cells were treated with filipin followed by rMGA_0676 (40 μg/ml) for 24 h at 37°C to detect the inhibition of rMGA_0676 internalization. The samples underwent IFA and were examined with a laser confocal scanning microscope. Cellular F-actin was stained with Alexa Fluor 555-conjugated phalloidin. The nuclei were counterstained with DAPI (blue). The rMGA_0676 was hybridized with mouse anti-His monoclonal antibody and labeled with FITC-conjugated goat anti mouse IgG antibody green fluorescence (GF). The normal DF-1 cells as the negative control (Con), cells were treated with Cholera toxin-FITC as the caveolin-mediated endocytic positive control (Cholera toxin). **(B)** The mean value of GF was used to quantify the positive staining for rMGA_0676 in filipin treated DF-1 cells **(A)**, the data were analyzed using SPSS software, and the graph was made using GraphPad Prism 5.0. “^**^”represented statistically significant differences (*P* < 0.01). **(C)** Internalization of rMGA_0676 was not blocked by clathrin-mediated endocytic inhibitor MDC. DF-1 cells were treated with MDC followed by rMGA_0676 (40 μg/ml) for 24 h at 37°C and detected the inhibition of rMGA_0676 internalization underwent IFA as described above. The normal DF-1 cells as the negative control (Con), cells were treated with transferrin as the clathrin-mediated endocytic positive control (transferrin). **(D)** The mean value of GF was used to quantify the positive staining for rMGA_0676 in MDC treated DF-1 cells **(B)**, the data were analyzed using SPSS software, and the graph was made using GraphPad Prism 5.0. **(E)** Internalization of rMGA_0676 was reduced by knockdown of caveolin in DF-1 cells by siRNA. The caveolin siRNA sequence(si Cave) and its negative control siRNA sequence(NC) were transfected into DF-1 cells using RNAiMAX reagent followed by rMGA_0676 (40 μg/ml) for 24 h at 37°C and detected the inhibition of rMGA_0676 internalization underwent IFA as described above. The normal DF-1 cells as the negative control (Con), cells transfected with negative control siRNA sequence followed by rMGA_0676 for 24 h at 37°C as the siRNA negative control (NC), cells were treated with Cholera toxin-FITC as the caveolin-mediated endocytic positive control (Cholera toxin). **(F)** The mean value of GF was used to quantify the positive staining for rMGA_0676 in caveolin siRNA sequence (Cave i) transfected DF-1 cells **(C)**, the data were analyzed using SPSS software, and the graph was made using GraphPad Prism 5.0. “^**^”represented statistically significant differences (*P* < 0.01), “ns” represented no significant differences. **(G)** Internalization of rMGA_0676 was not reduced by knockdown of clathrin in DF-1 cells by siRNA. The clathrin siRNA sequence(Clath i) and its negative control siRNA sequence(NC) were transfected into DF-1 cells using RNAiMAX reagent followed by rMGA_0676 (40 μg/ml) for 24 h at 37°C and detected the inhibition of rMGA_0676 internalization underwent IFA as described above. The normal DF-1 cells as the negative control (Con), cells transfected with negative control siRNA sequence followed by rMGA_0676 for 24 h at 37°C as the siRNA negative control (NC), cells were treated with transferrin as the clathrin-mediated endocytic positive control (transferrin). **(H)** The mean value of GF was used to quantify the positive staining for rMGA_0676 in clathrin siRNA sequence (Clath i) transfected DF-1 cells **(D)**, the data were analyzed using SPSS software, and the graph was made using GraphPad Prism 5.0. “^**^”represented statistically significant differences (*P* < 0.01), “ns” represented no significant differences. **(I)** Co-localization of MGA_0676 and caveolin in DF-1 cells. The sections were treated with rMGA_0676 (40 μg/ml) for 24 h at 37°C followed by IFA as described above. The Sections were incubated with mouse anti-His antibody and rabbit anti-caveolin antibody for 1.5 h at 37°C. Sections were then incubated with FITC-conjugated goat anti mouse IgG antibody (green) and rhodamine (tetramethyl rhodamine isocyanate [TRITC])-conjugated goat antirabbit antibody (red). Nuclei were counterstained with DAPI (blue). All the cell samples were examined with a laser confocal scanning microscope. Scale bar = 10 μm.

Bacterial toxins are endocytosed through caveolin-mediated or clathrin-dependent mechanisms. For instance, filipin can prevent caveolae vesicle formation while monodansylcadaverine (MDC) can inhibit clathrin-dependent vacuole formations (Orlandi and Fishman, 1998; Rejman et al., 2004; Veiga and Cossart, 2006; Kamashev et al., 2011). Therefore, we pretreated DF-1 cells with both filipin and MDC, and found that the internalization of rMGA_0676 was suppressed in a dose-dependent manner in cells pretreated with filipin (Figure S2A, Figures 1A,B), but pretreatment with MDC did not change the internalization of rMGA_0676 (Figures 1C,D). SiRNA technology was also employed to verify the above results. Figure S2C shows a reduction of caveolin in DF-1 cells treated with siRNA and a significant inhibition of the internalization of rMGA_0676 (Figure S2B). Inhibition of internalization can also be observed by confocal laser scanning microscopy (Figures 1E,F), from which rMGA_0676 and caveolin were found to be co-localizing in DF-1 cells (Figure 1I). The above results indicate that caveolin-mediated endocytosis is a dominant pathway for rMGA_0676 internalization. Clathrin was also silenced with siRNA, but no obvious effect on the internalization of rMGA_0676 was observed (Figures 1G,H).

### MGA_0676 interacts with NEDD8-activating enzyme (NAE)

**Figure 2:**
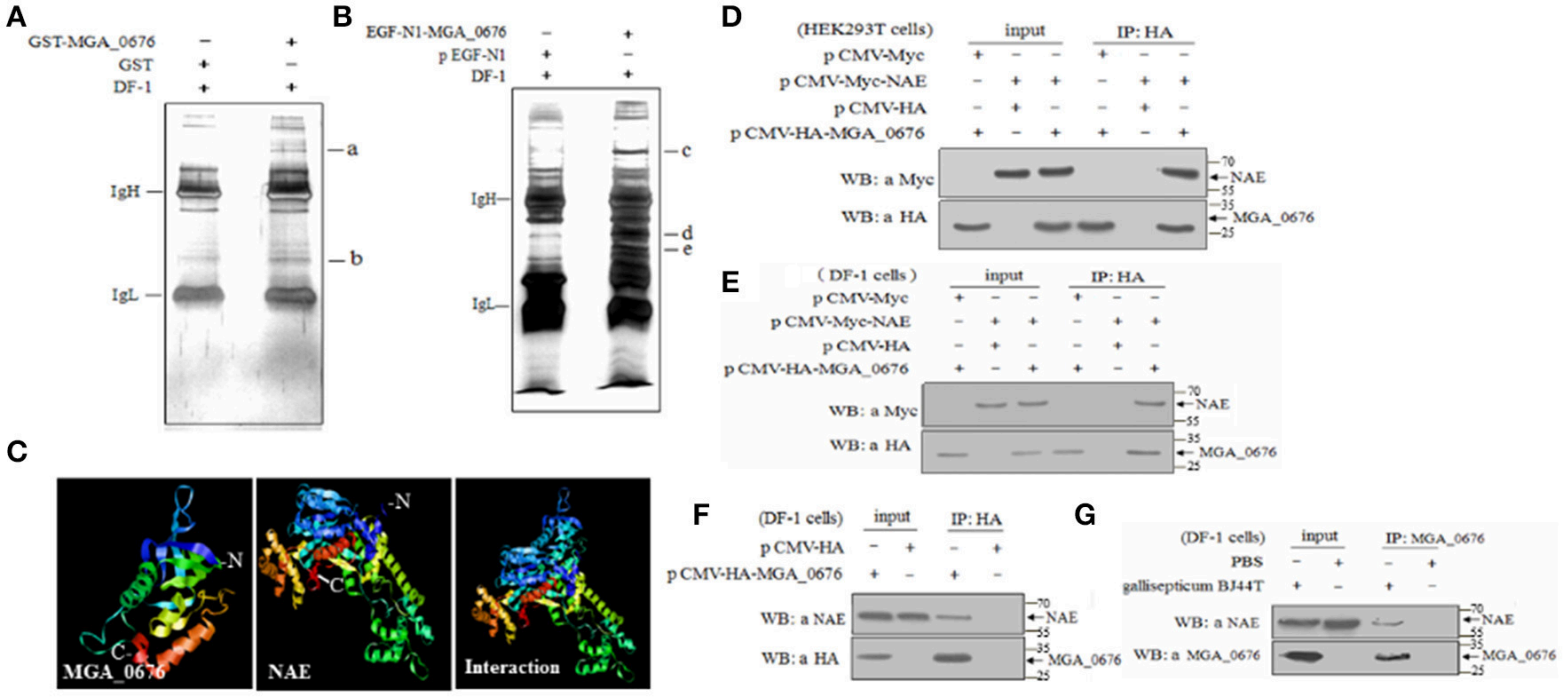
MGA_0676 interacts with NEDD8- activating enzyme (NAE). **(A)** Pull down assays based on GST. IgH and IgL indicate the heavy and light chains in the antibodies; a and b show the target proteins' interactions with MGA_0676. **(B)** Pull down assays based on GFP. IgH and IgL indicate the heavy and light chains in the antibodies; c, d, and e show the target proteins' interactions with MGA_0676. **(C)** A model of MGA_0676 binding to NAE. MGA_0676 can bind NAE through an interaction between the C-terminal of MGA_0676 and the N-terminal of NAE. **(D)** HEK293T cells were transfected with the indicated expression plasmids. **(E)** DF-1 cells were transfected with the indicated expression plasmids. Cell lysates were immunoprecipitated (IP) with anti-HA antibody and immunoblotted with anti-HA or anti-Myc antibodies. **(F)** Interaction of MGA_0676 with endogenous NAE. IP of cell lysates with anti-HA antibody, and immunoblotted with anti-HA or anti-NAE antibodies. **(G)** Interaction of *M. gallisepticum* MGA_0676 with endogenous NAE. DF-1 cells were infected with *M. gallisepticum* or PBS as a control. IP with anti-MGA_0676 antibody and immunoblotted with anti-MGA_0676 or anti-NAE antibodies.

In order to determine the target proteins of MGA_0676, GST fusion MGA_0676 protein was expressed and purified from recombinant *E. coli* (Figure S3A), and used as bait in the pull down system to screen proteins from DF-1 cells. Two proteins (a and b) were selected as candidate target proteins (Figure 2A). GFP-MGA_0676 expressed in DF-1 cells was also used to screen proteins from DF-1 cells by pull down, and three proteins (c, d, and e) were selected as candidate target proteins (Figure 2B). Mass spectrometry sequencing (MS) results of the above five proteins showed that NEDD8 activity enzyme E1 (NAE) chain B (structure Appbp1-Uba3-Nedd8-Mgatp-Ubc12) had the highest MS sequencing score, which is the most likely target protein interacting with MGA_0676 (Table 1).

**Table 1:** Proteins present in two specific bands were observed in the GST and GFP lanes as identified by MS.

**Bands**	**Accession no**.	**Protein name**	**Protein score**
a	gi|126031226	Chain B, Structure of Appbp1-Uba3-Nedd8-Mgatp-Ubc12	666
b	gi|84402	Glutathione transferase(fragment)	750
c	gi|126031226	Chain B, Structure of Appbp1-Uba3-Nedd8-Mgatp-Ubc12	403
d	F1NPP2	Uncharacterized protein	86
e	H9KZ27	Uncharacterized protein OS	91

In order to test the potential interaction, a model of the MGA_0676-NAE complex was constructed, analysis of the structure prediction models suggested that MGA_0676 could perfectly interact with NAE, and that the interacting domain should be located at the C-terminal of MGA_0676 and the N-terminal of NAE (Figure 2C). Furthermore, plasmids expressing HA-MGA_0676 or Myc-NAE were constructed to analyze their interactions within mammalian and chicken cells. When lysates of HEK293 T cells expressing both HA-MGA_0676 and Myc-NAE were immunoprecipitated with HA monoclonal antibody, Myc-NAE was detected in the precipitate, which indicates that MGA_0676 interacts with NAE ectopically expressed in mammalian cells (Figure 2D). Similar results were obtained in chicken DF-1 cells (Figure 2E), indicating that the interaction between the two proteins is not cell type specific. To confirm the interaction between the protein and host cells, DF-1 cells were transfected with pCMV-HA-MGA_0676 expression plasmid and an immunoprecipitation assay was performed with anti-HA monoclonal antibody. NAE was detected in the lysate of HA-MGA_0676-expressing cells after immunoprecipitation with anti-HA antibody (Figure 2F), which indicates that MGA_0676 interacts with endogenous NAE. DF-1 cells were then treated with *M. gallisepticum* BJ44T, lysed and immunoprecipitated with anti-MGA_0676 monoclonal antibody, and the precipitate was blotted with anti-NAE antibody. Both MGA_0676 and NAE were found in the precipitate (Figure 2G), indicating that *M. gallisepticum* MGA_0676 also interacted with endogenous NAE in host cells.

**Figure 3:**
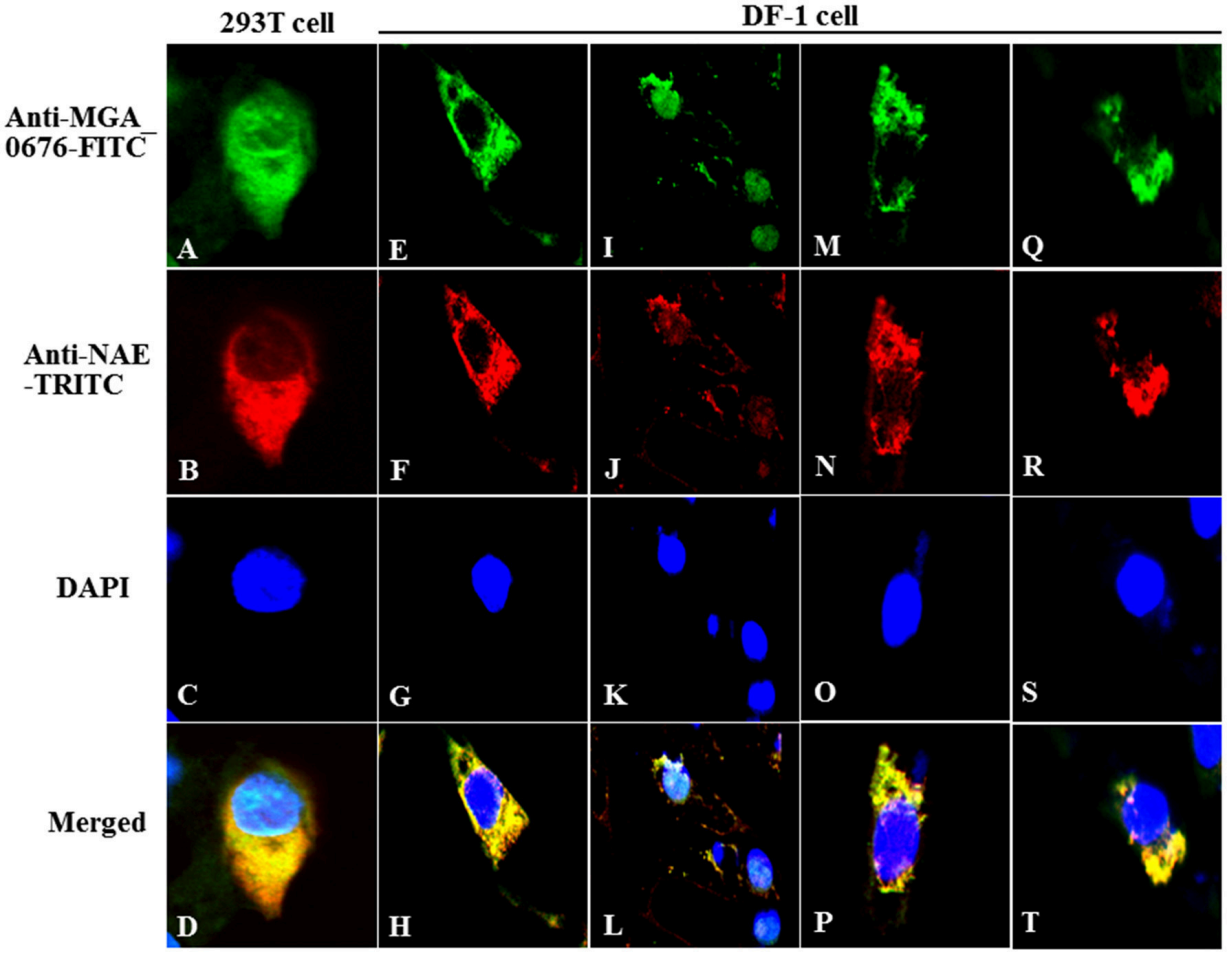
Co-localization of MGA_0676 with NAE in the perinuclear and nuclear regions of cells. HEK293T cells **(A–D)** or DF-1 cells **(E–G)** were co-transfected with pCMV-HA-MGA_0676 and pCMV-Myc-NAE. Twenty-four hours after transfection, cells underwent IFA based on anti-HA or anti-Myc antibodies. MGA_0676 was stained with FITC-conjugated goat anti mouse IgG antibody (green), NAE was stained with rhodamine (TRITC)-conjugated goat anti-rabbit antibody (red), and cell nuclei were counterstained with DAPI (blue). Cell samples were observed with a laser confocal scanning microscope. Co-localization of MGA_0676 with endogenous NA, DF-1 cells were transfected with pCMV-HA-MGA_0676 plasmid **(I–L**) or incubated with rMGA_0676 **(M–P**). Twenty-four hours after treatment, cells underwent IFA based on anti-HA or anti-NAE antibodies; cell samples were observed with a laser confocal scanning microscope. Co-localization of *M. gallisepticum* MGA_0676 with endogenous NAE in *M. gallisepticum*-infected cells **(Q–T**). DF-1 cells were infected with *M. gallisepticum*. Twenty-four hours after treatment, cells underwent IFA based on anti-MGA_0676 or anti-NAE antibodies; IFA and the following results were observed with a laser confocal scanning microscope. Scale bar = 10 μm.

Furthermore, the interaction between MGA_0676 and NAE was observed in HEK293T and DF-1 cells expressing HA-MGA_0676 and Myc-NAE by confocal microscopy, which shows HA-MGA_0676 and Myc-NAE co-localize in the perinuclear and nuclear regions of cells (Figures 3A–H). To determine whether endogenous NAE co-localizes in the same cellular compartment, DF-1 cells were transfected to express HA-MGA_0676 or treated with rMGA_0676, and a similar co-localization between HA-MGA_0676 and NAE was found (Figures 3I–P). DF-1 cells were also treated with *M. gallisepticum* and IFA was done using anti-MGA_0676 and anti-NAE antibodies. As expected, endogenous NAE also co-localizes with MGA_0676 in *M. gallisepticum*-infected cells (Figures 3Q–T).

### The SNC region of MGA_0676 interacts with the thif region of NAE

**Figure 4:**
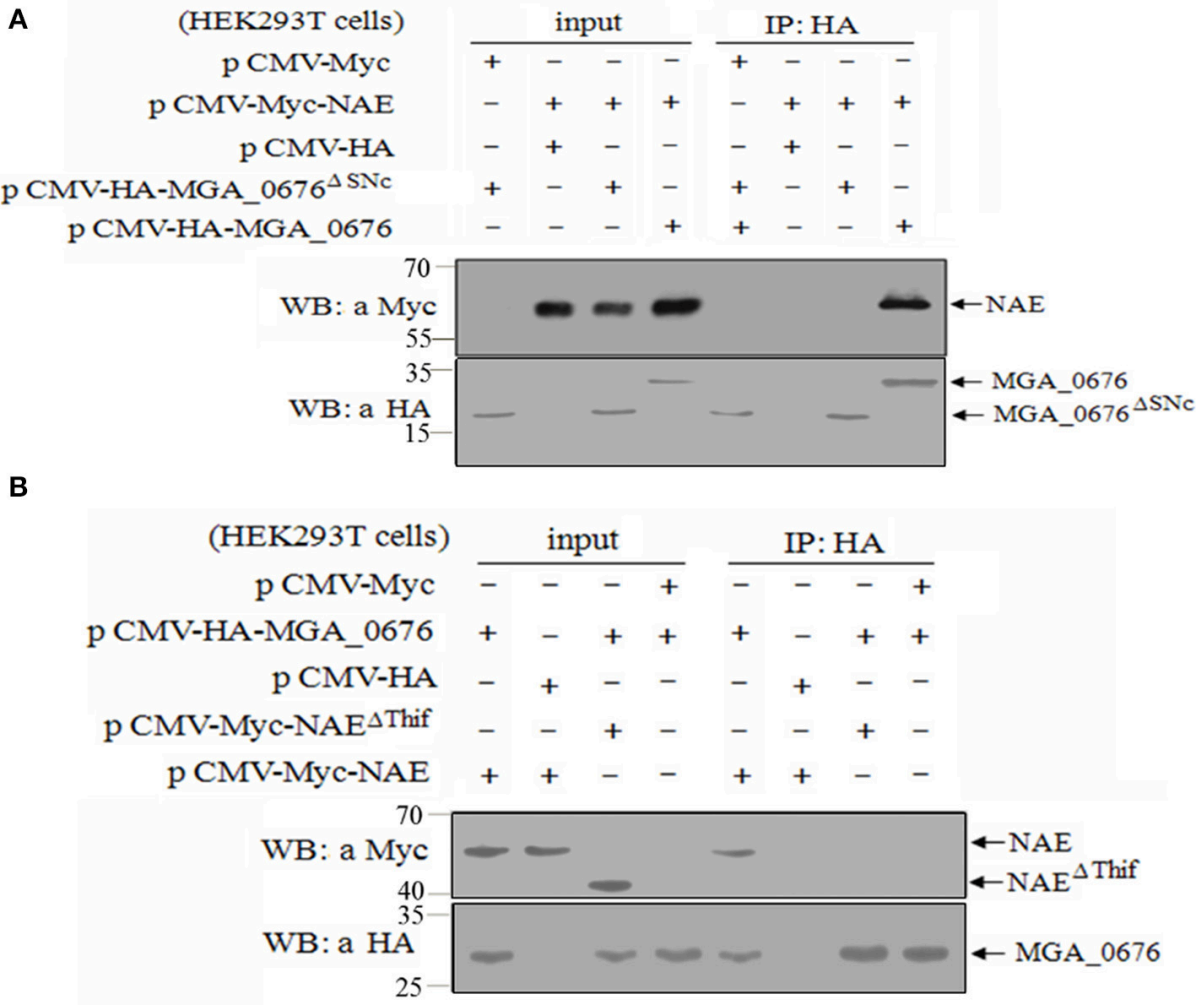
The SNC region of MGA_0676 interacts with the Thif region of NAE. **(A)** Endogenous NAE cannot interact with MGA_0676^Δ*SNC*^ (The SNC portion of MGA_0676, from amino acids 92 to 221 was deleted). HEK293T cells were transfected with full-length HA-MGA_0676 and HA-MGA_0676^Δ*SNC*^ molecules or empty vectors. Twenty-four hours after transfection, cell lysates were prepared and IP with anti-HA monoclonal antibody. The pellets were examined by western blotting using anti-Myc antibody. **(B)** Endogenous NAE^Δ*Thif*^ cannot interact with MGA_0676. HEK293T cells were transfected with full-length Myc-NAE and Myc-NAE^Δ*Thif*^ (The Thif region of NAE from amino acids 29 to 166 was deleted) molecules or empty vectors. Twenty-four hours after transfection, cell lysates were prepared and IP with anti-HA monoclonal antibody. The pellets were examined by western blotting using anti-Myc antibody.

To determine the region of MGA_0676 that interacts with NAE, a HA tag fused MGA_0676 mutant (MGA_0676^Δ*SNC*^) was constructed with a deleted SNC region that located at 92 to 221 amino acids of MGA_0676. The MGA_0676^Δ*SNC*^ derivative was expressed in HEK293T cells, and immunoprecipitation results indicated that MGA_0676^Δ*SNC*^ lost the ability to interact with NAE (Figure 4A). To determine the region of NAE that interacts with MGA_0676, a Myc tag fused NAE mutant (NAE^Δ*Thif*^) was constructed with a deleted Thif region that located at 29 to 166 amino acids of NAE. Immunoprecipitation results showed that the Thif region of NAE is necessary to interact with MGA_0676 (Figure 4B). The above results indicate that the SNC region of MGA_0676 interacts with the Thif region of NAE.

### MGA_0676 interacts with NAE to induce apoptosis in DF-1 cells

**Figure 5:**
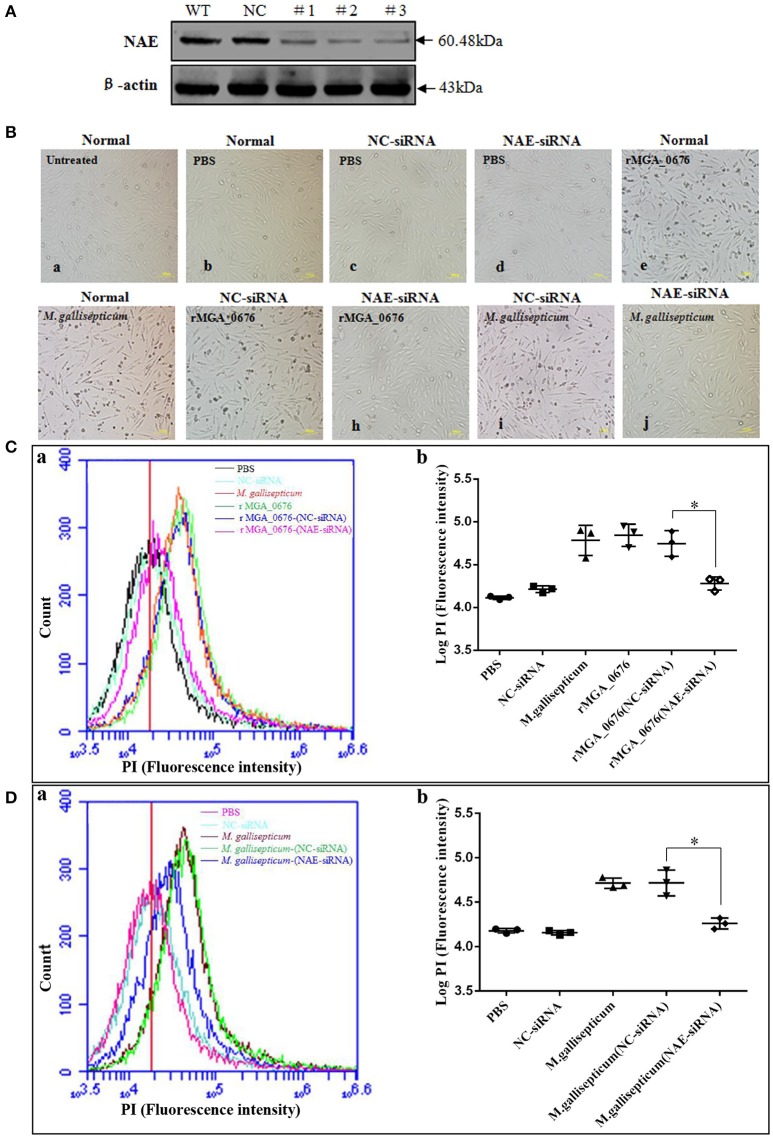
MGA_0676 interacts with NAE inducing apoptosis in DF-1 cells. **(A)** Effect of NAE RNAi on the expression of endogenous NAE. DF-1 cells were transfected with siRNA (#1 to #3) or controls (WT and NC), as described in Materials and Methods. Forty-eight hours after the second transfection, cell lysates were prepared and examined by western blotting with anti-NAE antibody. Endogenous β-actin expression was used as an internal control. **(B)** Pathological changes in NAE inhibited DF-1 cells after rMGA_0676 treatment. All cell groups were observed with a microscope after treatment as follows. a: normal cells; b: PBS-treated; c: siRNA NC; d: siRNA NAE; e: rMGA_0676 (40 μg/ml); f: *M. gallisepticum* (MOI = 10); g: siRNA NC pre-treatment, rMGA_0676 (40 μg/ml) treatment; h: siRNA NAE pre-treatment, rMGA_0676 (40 μg/ml) treatment; i: siRNA NC pre-treatment, *M. gallisepticum* (MOI = 10) treatment; j: siRNA NAE pre-treatment, *M. gallisepticum* (MOI = 10) treatment. Scale bar = 100 μm. **(C)** Apoptosis induction by rMGA_0676 in NAE arrested DF-1 cells. The curves **(a)** and the scatter plot **(b)** represent apoptotic cells in **(B)** treated groups. PBS (5Bb); NC-siRNA (5Bc); rMGA_0676 (5Be); *M. gallisepticum* (5Bf); rMGA_0676-(NC-siRNA) (5Bg); rMGA_0676-(NAE-siRNA) (5Bh). “^*^”represented statistically significant differences (*P* < 0.05). **(D)** Apoptosis induction by *M. gallisepticum* in NAE arrested DF-1 cells. The curves **(a)** and the scatter plot **(b)** also represent apoptotic cells in **(B)** treated groups. PBS (5Bb); NC-siRNA (5Bc); *M. gallisepticum* (5Bf); *M. gallisepticum*-(NC-siRNA) (5Bi); *M. gallisepticum*-(NAE-siRNA) (5Bj). “^*^”represented statistically significant differences (*P* < 0.05).

The binding of MGA_0676 to NAE suggests that NAE plays an important role in MGA_0676-induced apoptosis. When NAE of DF-1 cells was silenced via siRNA (Figure 5A), rMGA_0676 or *M. gallisepticum* did not induce obvious morphological changes (Figure 5B), rMGA_0676 did not induce observable apoptosis (Figure 5C), and the apoptosis of *M. gallisepticum*-infected cells decreased significantly (Figure 5D). The results of these experiments suggest that the interaction of NAE and MGA_0676 is related to the apoptosis of DF-1 cells induced by MGA_0676.

### Nuclear factor kappa B (NF-κB) is activated via interaction of MGA_0676 with NAE

**Figure 6:**
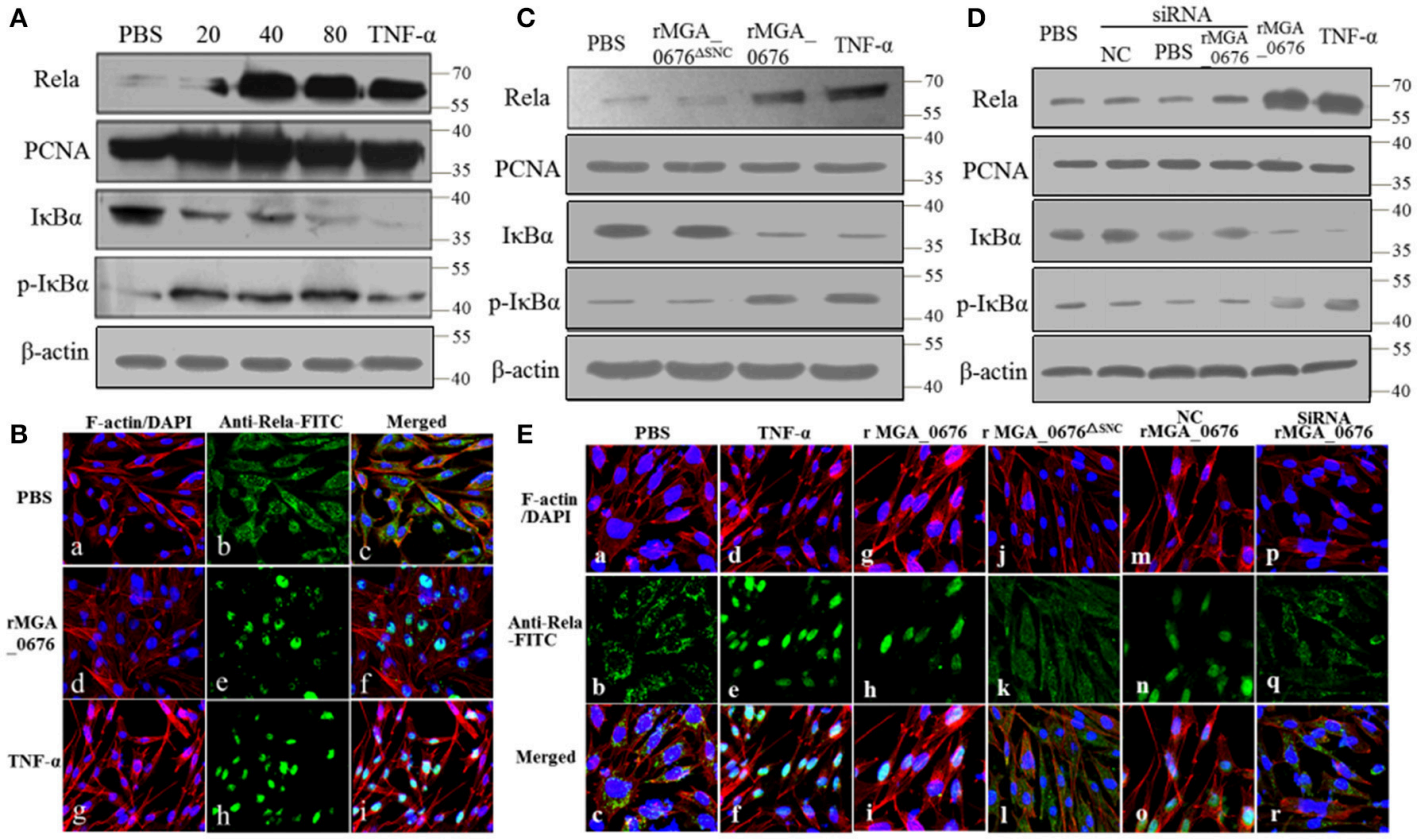
NF-κB is activated via interaction between MGA_0676 and NAE. **(A)** rMGA_0676 activated NF-κB in DF-1 cells were detected by western blot. The activity of NF-κB is rMGA_0676 dose dependent in DF-1 cells. DF-1 cells were treated with PBS or different doses of rMGA_0676 at 37°C. Twenty-four hours after transfection, cell lysates were prepared to detect the expression of Rela in the nucleus; PCNA was used as an internal control. Expression of IκBα and p-IκBα in the cytoplasm; β-actin expression was used as an internal control. **(B)** Translocation of Rela from the cytoplasm to the nucleus in DF-1 cells after rMGA_0676 treatment. **(a–c)** cells were treated with PBS as a negative control and then subjected to IFA based on anti-Rela antibody, followed by incubation with FITC-conjugated goat anti-mouse IgG antibody (green) and cellular F-actin staining with Alexa Fluor 555-conjugated phalloidin (red); nuclei were counterstained with DAPI (blue). The cell samples were observed with a laser confocal scanning microscope. **(d–f)** cells were treated with rMGA_0676 (40 μg/ml) for 24 h at 37°C; IFA and sample observation were done with a laser confocal scanning microscope. **(j–i)** cells were treated with TNF-α as a positive control. Scale bar = 50 μm. **(C)** NF-κB was not activated by MGA_0676^Δ*SNC*^. DF-1 cells were treated with PBS, rMGA_0676, or rMGA_0676^Δ*SNC*^; TNF-α was used as a positive control. After twenty-four hours, cell lysates were prepared to detect the expression levels of Rela, IKBα, and p-IkBα in the cells. PCNA was used as the nucleus internal control and β-actin was used as the cytoplasm internal control. **(D)** NF-κB was not activated by MGA_0676 in DF-1 cells after knockdown of NAE. DF-1 cells were pretreated with siRNA and then treated with rMGA_0676. Cells treated with PBS, NC, and siRNA were used as negative control; cells treated with rMGA_0676 or TNF-α were used as positive control. After 24 h, Rela, IκBα, and p-IκBα were detected in the cells as described above. **(E)** Rela was not translocated from the cytoplasm to the nucleus in DF-1 cells after disruption of the interaction between MGA_0676 and NAE. DF-1 cells were subjected to IFA as described in Materials and Methods. **(a–c)** cells treated with PBS were used as negative control; **(d–f)** cells treated with TNF-α were used as positive control; **(g–i)** cells were treated with rMGA_0676; **(j–l)** cells were treated with rMGA_0676^Δ*SNC*^: **(m–o)** cells were pretreated with siRNA (negative control) and then treated with rMGA_0676; **(p–r)** cells were pretreated with siRNA NAE and then treated with rMGA_0676.

The activity of nuclear factor kappa B (NF-κB) was examined by immunoblotting and laser scanning confocal microscopy to explore the biological effects of the interactions between MGA_0676 and NAE. Levels of NF-κB in the nucleus and of p-IκBα in the cytoplasm increased while IκBα in the cytoplasm decreased significantly after DF-1 cells were treated with rMGA_0676 (Figure 6A). In addition, Rela (p65 in mammalian cells) translocated from the cytoplasm to the nucleus in DF-1 cells treated with rMGA_0676 or TNF-α, as shown in Figure 6B. Furthermore, NF-κB was not activated in DF-1 cells treated with MGA_0676^Δ*SNC*^ (Figure 6C) or with MGA_0676 after knockdown of NAE (Figure 6D). Laser scanning confocal microscopy confirmed that NF-κB lost the ability to translocate to the nucleus in DF-1 cells treated with MGA_0676^Δ*SNC*^ and in rMGA_0676-treated DF-1 cells that had undergone NAE-knockdown (Figure 6E). The above results indicate that MGA_0676 activates NF-κB via interaction with NAE.

### MGA_0676 activates NF-κB through acceleration of cullin neddylation of DF-1 cells

**Figure 7:**
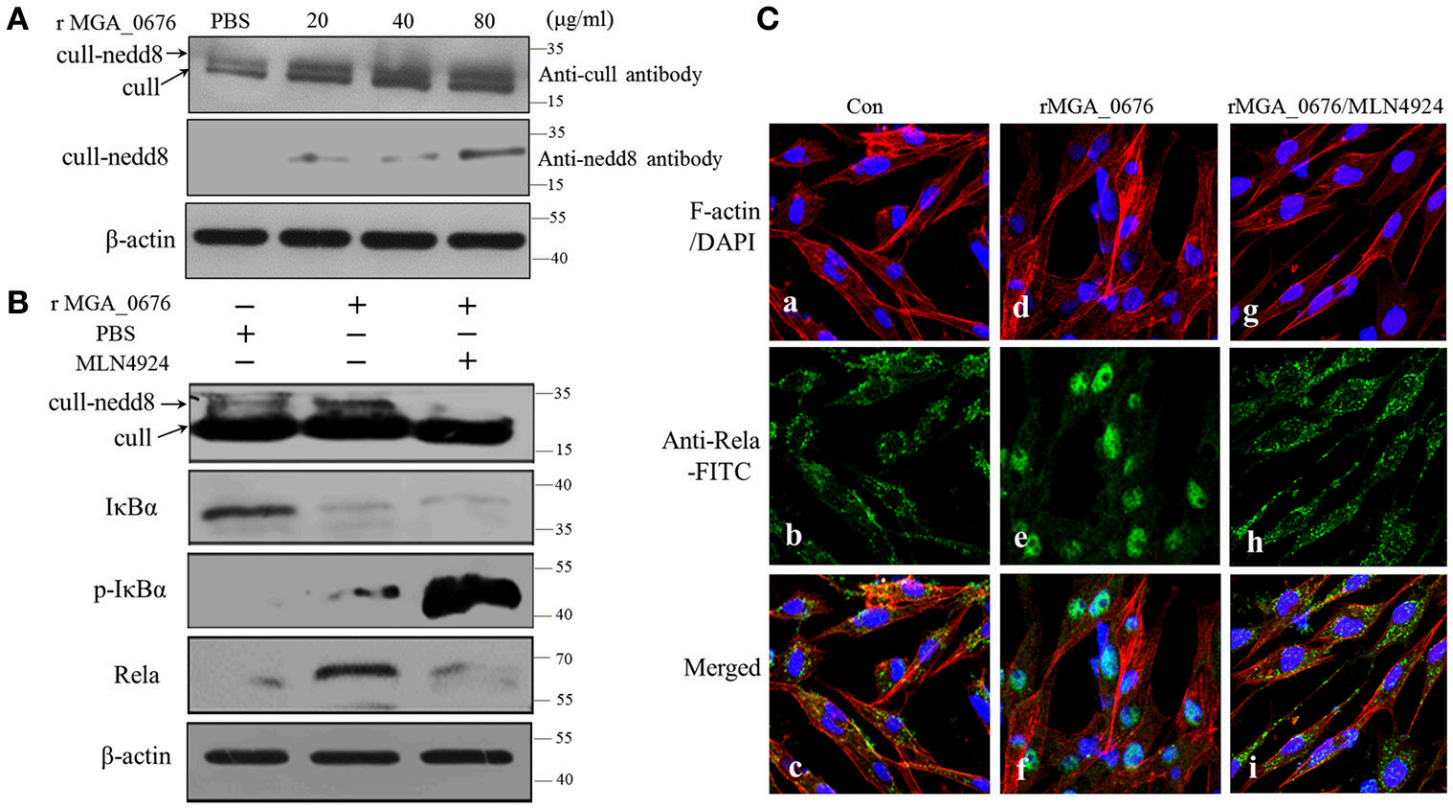
MGA_0676 activates NF-κB through accelerating the process of cullin neddylation in DF-1 cells **(A)** MGA_0676 accelerated the process of cullin neddylation in DF-1 cells. DF-1 cells were seeded on six-well plates and cultured for 24 h and then treated with rMGA_0676 at concentrations of 0, 20, 40, or 80 μg/ml for 24 h. Afterwards, cell lysates were prepared for western blot to detect the expression of cull-NEDD8 based on the anti-chicken cull antibody or anti-NEDD8 antibody. β-actin was used as internal reference. **(B)** Neddylation specific inhibitor MLN4924 blocked the activity of NF-kB induced by MGA_0676. DF-1 cells were seeded on six-well plates and cultured for 24 h and then treated with MLN4924 or MGA_0676 for 24 h. Afterwards, cell lysates were prepared to detect the expression of cull-NEDD8, IkBα, p-IkBα, and p65 (Rela) by western blot. β-actin was used as internal reference. **(C)** Translocation of Rela from the cytoplasm to the nucleus in DF-1 cells. **(a–c)** cells were treated with PBS as a negative control(Con); **(d–f)** cells were treated with rMGA_0676 (40 μg/ml) for 24 h at 37°C; **(g–i)** cells were treated with MLN4924 (500 ng/l) for 12–18 h at 37°C, then inclubated with rMGA_0676 (40 μg/ml) for 24 h at 37°C. All the cell samples were subjected to IFA based on anti-Rela antibody, followed by incubation with FITC-conjugated goat anti-mouse IgG antibody (green) and cellular F-actin staining with Alexa Fluor 555-conjugated phalloidin (red); nuclei were counterstained with DAPI (blue). The slices were observed with a laser confocal scanning microscope. Scale bar = 50 μm.

To explore the influence of cullin neddylation of DF-1 after treatment with MGA_0676, the cullin-NEDD8 complex of DF-1 cells was detected by western blot. As seen in Figure 7A, compared with the PBS treated DF-1 cells, expression of cullin-NEDD8 clearly increased in MGA_0676 treated DF-1 cells, which indicated that cullin neddylation of DF-1 was activated by MGA_0676. However, we also have shown above that MGA_0676 could activate NF-κB in DF-1 cells (Figure 6). For this reason, to investigate the possible interaction between neddylation and NF-κB in DF-1 cells after treatment with MGA_0676, we inhibited neddylation in DF-1 cells using a specific inhibitor, MLN4924. We found that neddylation specific inhibitor MLN4924 blocked the activation of NF-κB induced by MGA_0676 (Figure 7B), for further confirming this results, we observed the translocation of Rela in DF-1 cells by confocal microscopy, from the Figure 7C, we found that MGA_0676 lost its ability of inducing NF-κB translocated into nucleus when the DF-1 was pretreated with MLN4924. All these results demonstrated that MGA_0676 activated NF-κB through acceleration of cullin neddylation in DF-1 cells.

### MGA_0676 induces apoptosis of chicken embryo fibroblast by activating NF-κB

**Figure 8:**
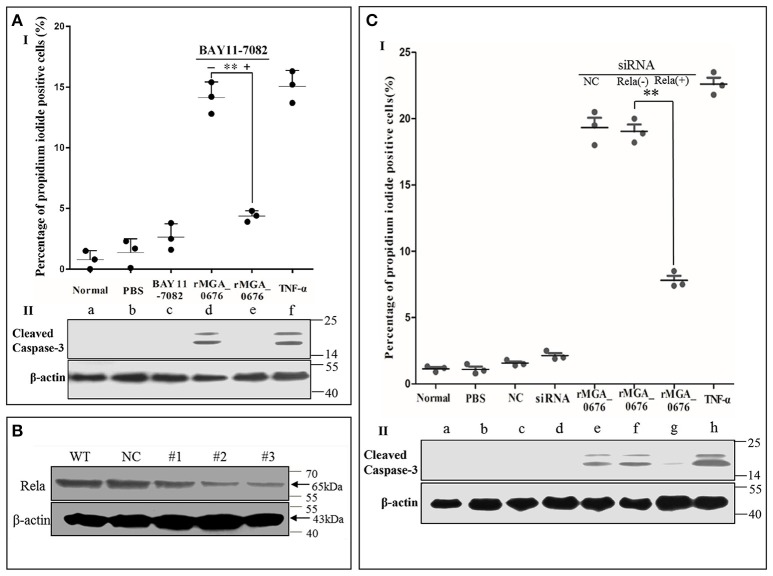
rMGA_0676 activates NF-κB inducing apoptosis in DF-1 cells. **(AI)** rMGA_0676-induced apoptosis in DF-1 cells was arrested by an NF-κB inhibitor. The cells were seeded on 12-well plates and cultured overnight. Apoptosis was assessed by the percentage of propidium iodide-positive cells following the treatment. a: normal cells; b: cells were treated with PBS; c: cells were treated with BAY 11-7082; d: cells were treated with rMGA_0676; e: cells were pretreated with BAY 11-7082 for 1 h at 37°C and then treated with rMGA_0676; f: cells were treated with TNF-α. The data were analyzed using SPSS software, and the graph was made using GraphPad Prism 5.0. “^**^”represented statistically significant differences (*P* < 0.01). **(AII)** Cleaved caspase 3 was detected in apoptosis-induced group (d and f). **(B)** Effect of Rela RNAi on the expression of endogenous Rela. DF-1 cells were transfected with siRNA (#1 to #3) or controls (WT and NC), as described in Materials and Methods. Forty-eight hours after the second transfection, cell lysates were prepared and examined by western blotting with anti-Rela antibody. Endogenous β-actin expression was used as an internal control. **(CI)** rMGA_0676-induced apoptosis in DF-1 cells was also arrested by knockdown of NF-κB. The cells were seeded on 12-well plates and cultured overnight, apoptosis was assessed by the percentage of propidium iodide positive cells following the treatment. a: normal cells; b: cells were treated with PBS; c: cells were treated with siRNA negative control (NC); d: cells were treated with siRNA NAE; e: cells were pretreated with siRNA NC for 48 h at 37°C and then treated with rMGA_0676; f: cells were treated with rMGA_0676; g: cells were pretreated with siRNA NAE for 48 h at 37°C and then treated with rMGA_0676; h: cells were treated with TNF-α. the data were analyzed using SPSS software, and the graph was made using GraphPad Prism 5.0. “^**^”represented statistically significant differences (*P* < 0.01). **(CII)** Cleaved caspase 3 was detected in apoptosis-induced group (e, f, and h).

Since we observed that the interaction between MGA_0676 and NAE activated NF-κB and was related to the apoptosis in DF-1 cells, we suspected NF-κB could play an important role during the apoptosis induced by MGA_0676. Therefore, in a subsequent experiment, we inhibited NF-κB with BAY 11-7082 (an inhibitor of NF-κB) and the apoptosis of DF-1 cells induced by MGA_0676 was examined. Figure 8AI and Figure S4 showed that the cell death phenotype treated with rMGA_0676 was significantly reduced by the addition of BAY 11-7082 (d and e). The key molecule of apoptosis cleaved caspase 3 was detected in apoptosis-induced group by western blot assays (Figure 8AII; d and f), and anti-cleaved caspase 3 antibodies and HRP-conjugated goat-anti-rabbit antibodies were used as primary and secondary antibodies, and the procedure was as described in “Material and Methods”. NF-κB was effectively silenced by siRNA (Figure 8B), and the apoptosis induced by rMGA_0676 decreased accordingly as shown by flow cytometry using PI staining (Figure 8CI, Figure S5), and cleaved caspase 3 was also detected in apoptosis-induced group (Figure 8CII; e, f, and h). These data strongly suggest that MGA_0676 induces apoptosis by activating NF-κB in DF-1 cells.

## Discussion

Endocytosis is involved in cell signaling generally mediated by clathrin (cytosolic protein) or caveolin (cholesterol-binding protein) (Pascual-Lucas et al., 2014; Haucke, 2015; Garcia et al., 2017). *Mycoplasma* nucleases have been found to be internalized host cells through endocytosis which are relevant to the process of infection (Schmidt et al., 2007; Li et al., 2010; Somarajan et al., 2010). Among of them, rMGA_0676 is a nuclease with a SNC region in *M. gallisepticum*, which can translocate into chicken cells and induce cell apoptosis, and MGA_0676 plays a important role in the process of infection of *M. gallisepticum* (Xu et al., 2015). In the present study, we show that endocytosis is the major process by which rMGA_0676 translocate into cells, and that it is mediated by caveolin (Figure S2, Figure 1). Unlike clathrin-dependent endocytosis, caveolar endocytosis has an important function in the cellular uptake of some bacterial toxins, viruses and circulating proteins, and it is usually mediated by the cellular receptors in caveolae of the host cells (Chaudhary et al., 2014; Schmidt et al., 2015). This implies there should be receptors of MGA_0676 on the membrane of chicken cells worthy of being identified.

Several nucleases (MPN133, MG_186, or mhp379, among others) have been discovered in *Mycoplasma*, and all of them are membrane-associated proteins that can induce apoptosis of host cells. These nucleases are thought to contribute to the pathological damage, immune evasion, and persistent infection of the host cells (Jones and Falkow, 1996; Schmidt et al., 2007; Li et al., 2010; Somarajan et al., 2010). NEDD8-activating enzyme (NAE) has been proposed to be an important member of the neddylation pathway in the cell cycle (Chen et al., 2000; Soucy et al., 2009). Furthermore, bacterial effector proteins can lead to ubiquitin/NEDD8 dysfunction via glutamine deamidation and induce macrophage-specific apoptosis by arresting the activity of NAE in host cells (Munro et al., 2007; Cui et al., 2010; Jubelin et al., 2010). In our study, we show that the apoptosis of DF-1 cells induced by rMGA_0676 decrease significantly after knockdown of endogenous NAE, suggesting that NAE is a crucial factor in MGA_0676-induced apoptosis. Moreover, MGA_0676 is found to interact with NAE by their co-location in the perinuclear and nuclear regions of DF-1 cells (Figures 2, 3), and the interaction happens between the SNC region of MGA_0676 and the Thif domain of NAE (Figure 4). The most important is the interaction of NAE and MGA_0676 is related to the apoptosis of DF-1 cells induced by MGA_0676 (Figure 5), which indicates the neddylation pathway maybe associated with apoptosis of DF-1 cells induced by MGA_0676.

NF-κB (nuclear factor kappa-light-chain-enhancer of activated B cells) is one of the first responders to harmful cellular stimuli since it belongs to the category of “rapid-acting” primary transcription factors. The activation of NF-κB may induce or inhibit apoptosis depending on the cell type and context of the stimulus (Chen et al., 2003; Brasier, 2006; Huang et al., 2015). Several mycoplasmal lipoproteins have been proven to induce apoptotic and necrotic cell death in lymphocytes and monocytes/macrophages through TLR2-mediated signaling, which leads to activation of NF-κB (Into et al., 2004; Wu et al., 2008), *M. gallisepticum* lipid associated membrane proteins through an NF-κB dependent pathway up-regulate inflammatory genes in chicken tracheal epithelial cells (Majumder et al., 2014). Similar activation of NF-κB and induction of apoptosis are found in our studies, which implies that toll-like receptors that recognize pathogen-associated molecules may be involved in the pathogenic process of MGA_0676 (Lee et al., 2014; Okumura and Nizet, 2014). Additional results in our study indicated that the activation of NF-κB is dependent on the interaction between MGA_0676 and NAE in DF-1 cells (Figure 6), and for this reason the exact molecular mechanism is also explored.

MGA_0676 activated NF-κB by accelerating the process of cullin neddylation in DF-1 cells (Figure 7), while the activation of NF-κB induced apoptosis of DF-1 cells (Figure 8). These findings indicated a relevant link between neddylation and NF-κB in chicken embryo fibroblasts after treatment by MGA_0676. These results were also the first to show the apoptosis induction mechanism of mycoplasmal nuclease. In addition, our results indicated that MGA_0676 may be an important etiological virulence factor of the respiratory disease caused by *M. gallisepticum*, and that it may be involved in the immunosuppression of the infected birds. MGA_0676 may also participate in the evasion from the immune clearance by the host, due to its ability of inducing apoptosis, which was probably mediated through the activation of NF-κB (Adegboye, 1978; Papayannopoulos, 2014). Knock-out of MGA_0676 or SNC region may decrease the virulence of *M. gallisepticum*, and the attenuated strain could be used to prepare vaccines to prevent the infection caused by *M. gallisepticum* in the future. In conclusion, in our study we found that the mycoplasmal nuclease MGA_0676 interacted with NAE and accelerated cullin neddylation, which activated the NF-κB to induce apoptosis of chicken embryo fibroblasts, but how this mechanism plays its vital role in the *M. gallisepticum* growth and infections remain further explored.

## Ethics statement

All animal care procedures and experiments were approved by the Beijing Association for Science and Technology (approval ID SYXK (Beijing) 2007-0023) and were in compliance with the Beijing Laboratory Animal Welfare and Ethics Guidelines issued by the Beijing Administration Committee of Laboratory Animals. All animal studies were performed in accordance with the China Agricultural University Institutional Animal Care and Use Committee Guidelines (ID: SKLAB-B-2010-003) and were approved by the Animal Welfare Committee of China Agricultural University.

## Author contributions

WW and JX conceived and designed the experiments. PL, JX, HR, XL, and YZ performed the experiments. WW and JX analyzed the data. FJ contributed reagents, materials, analysis tools. PL, JX and WW wrote the paper.

### Conflict of interest statement

The authors declare that the research was conducted in the absence of any commercial or financial relationships that could be construed as a potential conflict of interest.
