# Supplementary material for: Mechanism of Apoptosis Induction by Mycoplasmal Nuclease MGA_0676 in Chicken Embryo Fibroblasts
Source: Front Cell Infect Microbiol. 2018 Apr 4;8:105. doi: 10.3389/fcimb.2018.00105 (PMC5893762; doi:10.3389/fcimb.2018.00105)
Supplement: Supplementary file 1 [file Table1.PDF]

Table S1 Oligonucleotide primers used for amplification of NAE gene assembly and structurally related compounds

Number	Primer sequence (5’to 3’)[a]	Position(size in bp)[b]
**1. NAE**		
NAEF	GGATCCATGGCGCAGCCGGGCAGAG	1→19(19)
NAER	CTCGAGCTACAACTGGAAAGTGGCTGACGT	1585←1608(24)
**2. NAE$^{\Delta Thif}$**		
NAEUF	GGATCCATGGCGCAGCCGGGCAGAG	1→19(19)
NAEUR	CTGGATGCTCCTTGATTAAAGCTTCTTGTCCAT	71←87(17),499←515(16)
NAEDF	ATGGACAAGAAGCTTTAATCAAGGAGCATCCAG	71→87(14),499→515(16)
NAEDR	CTCGAGCTACAACTGGAAAGTGGCTGACGT	1585←1608(24)

a. Underline indicates introduced restriction enzyme sites. **b.** Nucleotide position within MGA_0676 coding open reading frame (ORF).

By way of illustration, Table S1 shows that the oligonucleotide primers are divided into NAE and NAE$^{\Delta Thif}$ groups, respectively.

Table S2 RNA oligonucleotides used for depletion of cells' genes in this study.

Number	SiRNA sequence (5'to 3')
**Negative control(NC)**	
Sense	UUCUCCGAACGUGUCACGUTT
Anti sense	ACGUGACACGUUCGGAGAATT
**1. NAE**	
RNAi#1	
Sense	GCAACACAGCUAUCGGAAATT
Anti sense	UUUCCGAUAGCUGUGUUGCTT
RNAi#2	
Sense	GCUGAUCUGUAGGACUUAUTT
Anti sense	AUAAGUCCUACAGAUCAGCTT
RNAi#3	
Sense	GGAUUGUGAUUGUUGCCAATT
Anti sense	UUGGCAACAAUCACAAUCCTT
**2.Caveolin(CAV)**	
RNAi#1	
Sense	GGGAACAGGGCAACAUCUATT
Anti sense	UAGAUGUUGCCCUGUUCCCTT
RNAi#2	
Sense	UCAACGACGACGUGGUGAATT
Anti sense	UUCACCACGUCGUCGUUGATT
RNAi#3	
Sense	AGAAGGAACACACAGCUUUTT
Anti sense	AAAGCUGUGUGUUCCUUCUTT
**3.Clathrin(CLTC)**	
RNAi#1	
Sense	GCAUCAACCCAGCAAACAUTT
Anti sense	AUGUUUGCUGGGUUGAUGCTT
RNAi#2	
Sense	GCUAGCACUUAGUGUCUAUTT
Anti sense	AUAGACACUAAGUGCUAGCTT
RNAi#3	
Sense	GCGUGAGAAUCCUUACUAUTT
Anti sense	AUAGUAAGGAUUCUCACGCTT
**4. NF-κB(ReLa)**	
RNAi#1	
Sense	CCAUCCGCGUAAACCAUUATT
Anti sense	UAAUGGUUUACGCGGAUGGTT
RNAi#2	
Sense	CGUGCACAGUUUCCAGAAUTT
Anti sense	AUUCUGGAAACUGUGCACGTT
RNAi#3	
Sense	CCCAGCCCAUCUAUGACAATT
Anti sense	UUGUCAUAGAUGGGCUGGGTT
