# Supplementary material for: Mechanism of Apoptosis Induction by Mycoplasmal Nuclease MGA_0676 in Chicken Embryo Fibroblasts
Source: Front Cell Infect Microbiol. 2018 Apr 4;8:105. doi: 10.3389/fcimb.2018.00105 (PMC5893762; doi:10.3389/fcimb.2018.00105)
Supplement: Supplementary file 2 [file Image1.PDF]

Figure S1 rMGA_0676 internalization was dose (A) and time (B) dependent in DF-1 cells

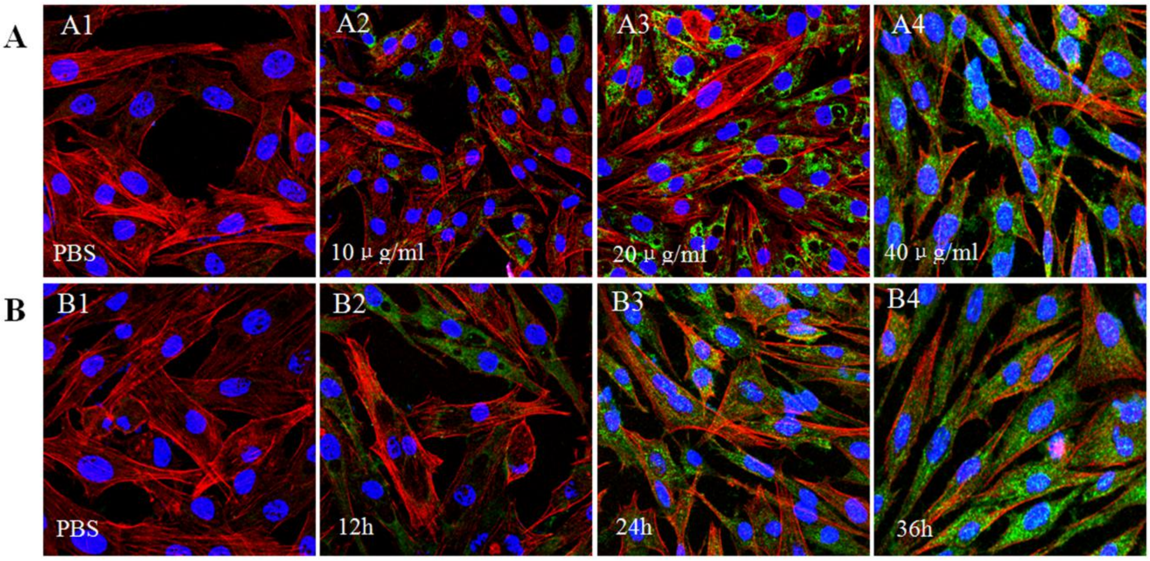

A. rMGA_0676 internalization was dose dependent in DF-1 cells. DF-1 cells were treated with different doses of rMGA_0676 for 24 h at 37°C. A1: PBS; A2: 20 μg/ml; A3: 40 μg/ml; A4: 80 μg/ml

B. rMGA_0676 internalization is time-dependent in DF-1 cells. DF-1 cells were treated with rMGA_0676 (40 μg/ml) at different time points at 37°C. B1: PBS; B2: 12 h; B3: 24 h; B4: 36 h.

Figure S2 Pretreatment of DF-1 cells with endocytic inhibitors (A) and small interference RNA (B and C)

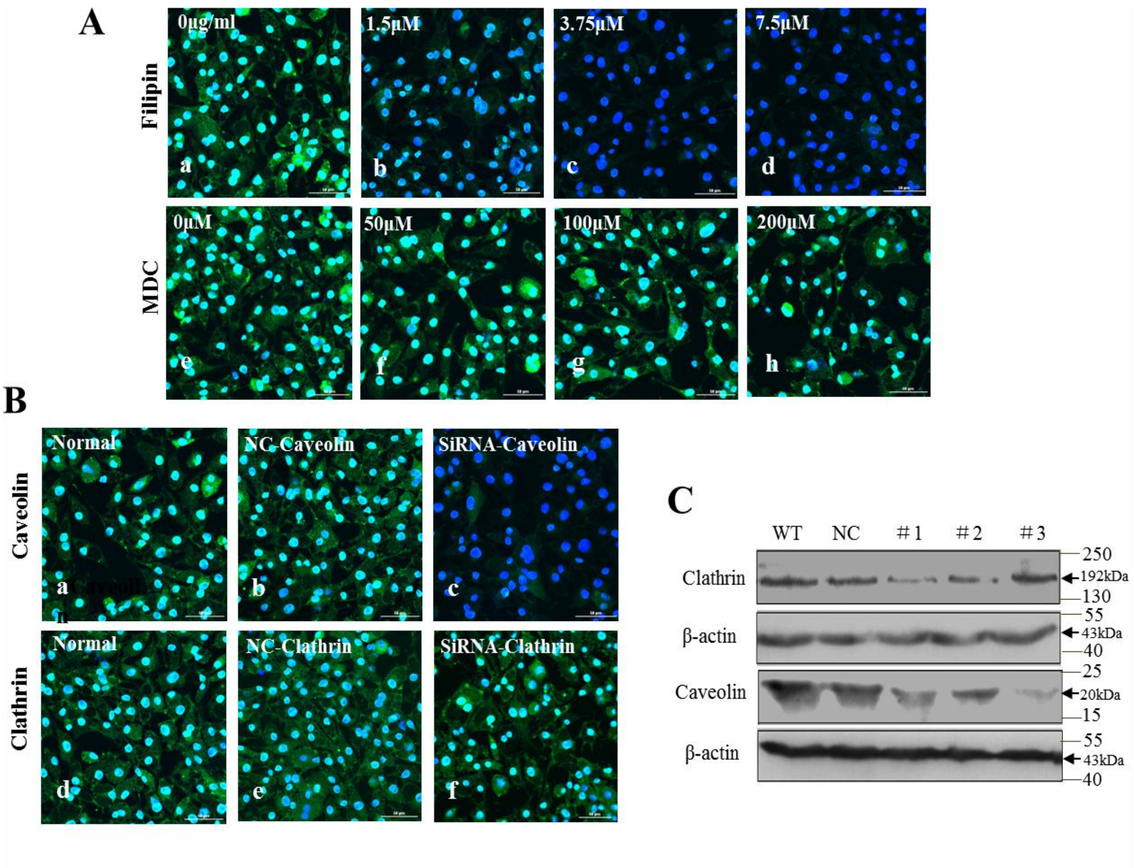

A: Internalization of rMGA_0676 in DF-1 cells was inhibited, in a dose-dependent manner, by the endocytic inhibitor filipin. DF-1 cells were pretreated with endocytic inhibitors for 1 h at 37°C, rMGA_0676 (40 μg/ml) was then added; the cells were incubated for 24 h and then subjected to IFA based on anti-MGA_0676 (green); the nuclei were counterstained with DAPI (blue) and the cell samples were observed with a laser confocal scanning microscope. a: rMGA_0676; b: filipin (1.5μM) and rMGA_0676; c: filipin (3.75 μM) and rMGA_0676; d: filipin (7.5 μM) and rMGA_0676; e: rMGA_0676; f: MDC (50 μM) and rMGA_0676; g: MDC (100 μM) and rMGA_0676; h: MDC (200 μM) and rMGA_0676.

B: Internalization of rMGA_0676 was inhibited by siRNA caveolin in DF-1 cells. DF-1 cells were pretreated with siRNA for 48 h at 37°C. rMGA_0676 (40 μg/ml) was then added; cells were incubated for 24 h and then subjected to IFA, as described above. a: rMGA_0676; b: caveolin siRNA negative control (NC-Caveolin) and rMGA_0676; c: siRNA caveolin (SiRNA-caveolin) and rMGA_0676; d: rMGA_0676; e: clathrin siRNA negative control (NC-Clathrin) and rMGA_0676; f: siRNA clathrin (siRNA-clathrin) and rMGA_0676.

C: Effect of clathrin or caveolin RNAi on the expression of endogenous clathrin or caveolin. DF-1 cells were transfected with siRNA (#1 to #3) or controls (WT and NC) as described in Materials and Methods. Forty-eight hours after the second transfection, cell lysates were prepared and examined by western blotting with anti-clathrin or caveolin antibodies. Endogenous β-actin expression was used as an internal control.

Figure S3 Expression of rMGA_0676 in E. coli (GST fused protein) and in DF-1 cells (GFP fused protein)

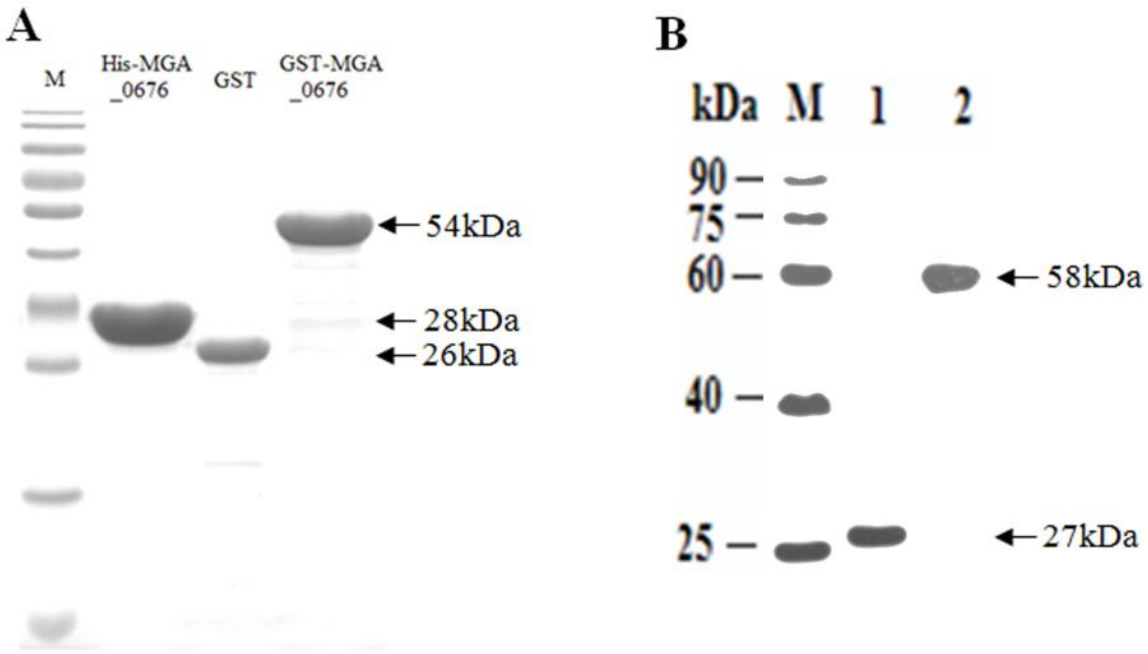

A: GST fused recombinant MGA_0676 was expressed and purified in *E. coli*. His-MGA0676: positive control; GST: GST labeled control; GST-MGA_0676: purified GST fused with the MGA_0676 protein.

B: GFP fused recombinant MGA_0676 was expressed in DF-1 cells. The pEGF-N1-MGA_0676 plasmids were transfected as described in Materials and Methods. Cell lysates were immunoblotted with anti-GFP antibody to detect the expression of GFP or GFP fused MGA_0676.

M: Molecular weight markers; 1: DF-1 cells transfected with pEGF-N1 vector; 2: DF-1 cells transfected with pEGF-N1-MGA_0676 vector.

Figure S4 rMGA_0676-induced apoptosis in DF-1 cells was arrested by an NF-κB inhibitor

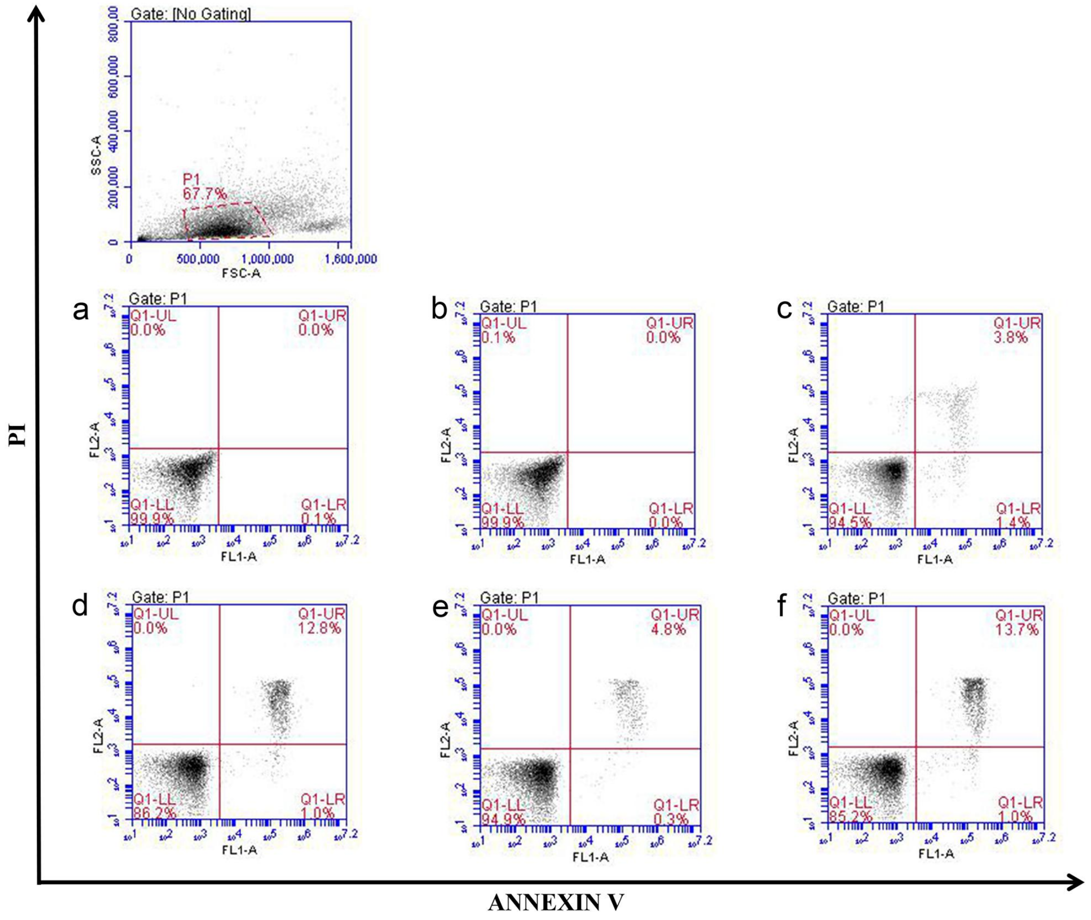

The cells were seeded on 12-well plates and cultured overnight. Apoptosis was assessed by the percentage of propidium iodide-positive cells following the treatment. **a:** normal cells; **b:** cells were treated with PBS; **c:** cells were treated with BAY 11-7082; **d:** cells were treated with rMGA_0676; **e:** cells were pretreated with BAY 11-7082 for 1 h at 37°C and then treated with rMGA_0676; **f:** cells were treated with TNF-α. The data were analyzed using SPSS software.

Figure S5 rMGA_0676-induced apoptosis in DF-1 cells was arrested by knockdown of NF-κB

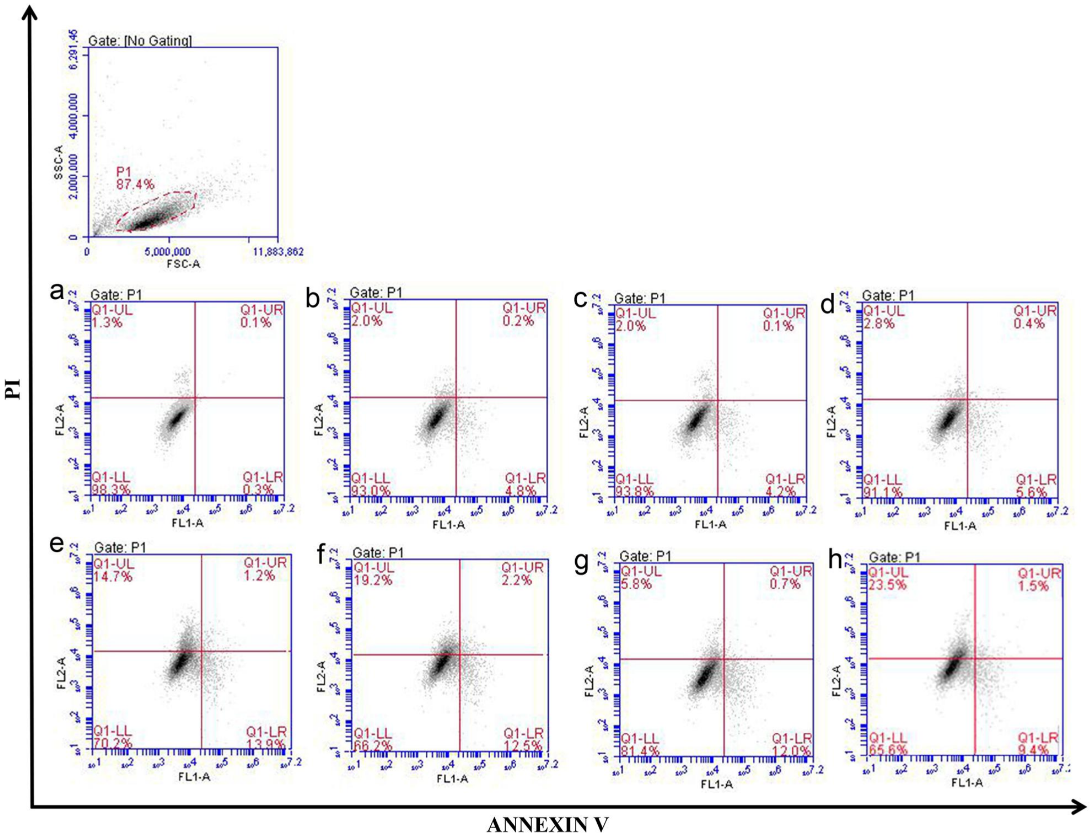

The cells were seeded on 12-well plates and cultured overnight, apoptosis was assessed by the percentage of propidium iodide positive cells following the treatment. **a:** normal cells; **b:** cells were treated with PBS; **c:** cells were treated with siRNA negative control (NC); **d:** cells were treated with siRNA NAE; **e:** cells were pretreated with siRNA NC for 48 hours at 37°C and then treated with rMGA_0676; **f:** cells were treated with rMGA_0676; **g:** cells were pretreated with siRNA NAE for 48 hours at 37°C and then treated with rMGA_0676; **h:** cells were treated with TNF-α. the data were analyzed using SPSS software.
